# 1,1′-[*m*-Phenyl­enebis(nitrilo­methanylyl­idene)]dinaphthalen-2-ol–1,1′-[*m*-phenyl­enebis(imino­methanylyl­idene)]dinaphthalen-2(1*H*)-one (0.58/0.42)

**DOI:** 10.1107/S1600536811040918

**Published:** 2011-10-12

**Authors:** Anita Blagus, Branko Kaitner

**Affiliations:** a Department of Chemistry, J.J. Strossmayer University, Osijek, Franje Kuhača 20, HR-31000 Osijek, Croatia; b Laboratory of General and Inorganic Chemistry, Department of Chemistry, Faculty of Science, University of Zagreb, Horvatovac 102a, HR-10002 Zagreb, Croatia

## Abstract

In the solid state the title Schiff base, 0.58C_28_H_20_N_2_O_2_·0.42C_28_H_20_N_2_O_2_, exists both as the keto–imino and as the enol–amino tautomer, which is manifested in the disorder of the H atom in the intra­molecular hydrogen-bonded ring. The naphthalene ring systems show some distortion, which is consistent with the quinoid effect. The ratio of the enol form refined to 58 (5)%. The mol­ecule has crystallographically imposed symmetry: a twofold axis passes through the central benzene ring. Crystals are built up of layers parallel to (010). Stacking interactions between the layers involve only standard van der Waals attraction forces between apolar groups. The alignment of the aromatic rings in neighbouring layers shows a herringbone motif. A weak C—H⋯O inter­action is observed.

## Related literature

For general background to Schiff bases, see: Blagus *et al.* (2010 ▶). For applications of Schiff bases and derivatives as ligands, see: Hernández-Molina *et al.* (1997 ▶); Torayama *et al.* (1997 ▶); Elerman *et al.* (1998 ▶); Ganjali *et al.* (2008 ▶). For discussion of the quinoid effect, see: Gavranić *et al.* (1996 ▶, 1997) ▶; Friščić *et al.* (1998 ▶). For structures with a herringbone arrangement, see: Desiraju & Gavezzotti (1989 ▶). For standard bond lengths, see: Allen *et al.* (1987 ▶).
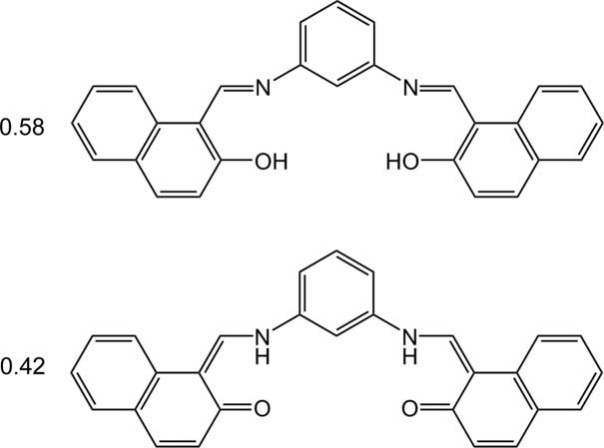


## Experimental

### 

#### Crystal data

0.58C_28_H_20_N_2_O_2_·0.42C_28_H_20_N_2_O_2_
*M* *_r_* = 416.46
Orthorhombic, 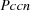

*a* = 5.4292 (9) Å
*b* = 26.496 (3) Å
*c* = 14.818 (2) Å
*V* = 2131.6 (5) Å^3^
*Z* = 4
Mo *K*α radiation
μ = 0.08 mm^−1^
*T* = 298 K
0.60 × 0.50 × 0.20 mm

#### Data collection

Oxford Diffraction Xcalibur CCD diffractometer
11523 measured reflections
2089 independent reflections
1699 reflections with *I* > 2σ(*I*)
*R*_int_ = 0.035

#### Refinement

*R*[*F* ^2^ > 2σ(*F* ^2^)] = 0.059
*wR*(*F* ^2^) = 0.166
*S* = 1.11
2089 reflections
153 parameters
1 restraint
H atoms treated by a mixture of independent and constrained refinement
Δρ_max_ = 0.18 e Å^−3^
Δρ_min_ = −0.15 e Å^−3^

### 

Data collection: *CrysAlis CCD* (Oxford Diffraction, 2003 ▶); cell refinement: *CrysAlis RED* (Oxford Diffraction, 2003 ▶); data reduction: *CrysAlis RED*; program(s) used to solve structure: *SHELXS97* (Sheldrick, 2008 ▶); program(s) used to refine structure: *SHELXL97* (Sheldrick, 2008 ▶); molecular graphics: *ORTEP-3* (Farrugia, 1997 ▶); software used to prepare material for publication: *WinGX* (Farrugia, 1999 ▶), *PARST97* (Nardelli, 1995 ▶) and *Mercury* (Macrae *et al.*, 2006 ▶).

## Supplementary Material

Crystal structure: contains datablock(s) I, global. DOI: 10.1107/S1600536811040918/fy2025sup1.cif

Structure factors: contains datablock(s) I. DOI: 10.1107/S1600536811040918/fy2025Isup2.hkl

Additional supplementary materials:  crystallographic information; 3D view; checkCIF report

## Figures and Tables

**Table 1:** Hydrogen-bond geometry (Å, °)

*D*—H⋯*A*	*D*—H	H⋯*A*	*D*⋯*A*	*D*—H⋯*A*
O1—H1⋯N1	0.96	1.66	2.569 (2)	154
N1—H2⋯O1	0.99	1.82	2.569 (2)	130
C13—H13⋯O1^i^	0.93	2.67	3.413 (3)	137

Symmetry code: (i) 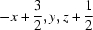.

**Fig. 1.**
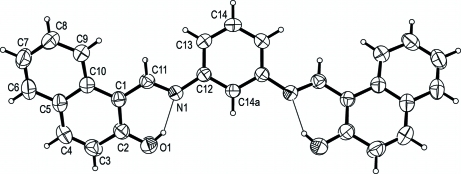
ORTEP-3 view of the enol tautomer of 1,3-bis(2-hydroxy-1-naphthylmethylideneamino)benzene with anisotropic thermal ellipsoids drawn at the 50% probability level. Only symmetry independent atoms are labelled.

**Fig. 2.**
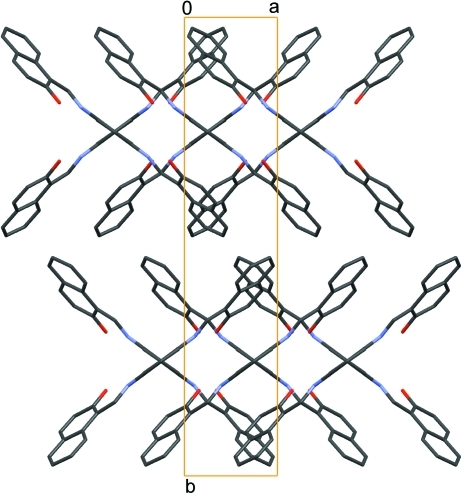
Packing diagram projected down the c-axis showing the herringbone motif between layers stacked along the b-axis.

## supplementary crystallographic information

### Comment

Schiff bases are among the most fundamental chelating systems in coordination chemistry. They have been used extensively as ligands with numerous transition and *p*-block metals (Blagus *et al.*, 2010). The conformation of the free ligand is of interest for comparison with the coordinated one in a corresponding metal complex (Gavranić *et al.*, 1997). Schiff bases derived from *m*-phenylenediamine can coordinate only one of their two ligand nitrogen atoms to a particular metal cation, thereby facilitating the formation of binuclear complexes, in which two coordination centres are bridged by two Schiff base molecules (Hernández-Molina, *et al.*, 1997; Torayama *et al.*, 1997). The environment of the coordination centre in transition metal complexes with bidentate Schiff base ligands can be modified by attaching different substituents to the ligand. Substituents have been used for tuning both steric and electronic properties, which are important for differences in structure and reactivity (Elerman *et al.*, 1998; Ganjali *et al.*, 2008).

The title compound is a stretched, non-planar aromatic system consisting of five aromatic and two hydrogen-bonded pseudo-aromatic rings with certain possibility of electron flow through the system (Fig. 1). The molecules are present in the crystals as disordered keto- and enol tautomers in an approximate ratio of 42 (5) : 58 (5).

The shape of the molecule can be conveniently described *via* three best planes, calculated through the two terminal naphthalene fragments and the central benzene ring. Because of the imposed crystallographic twofold symmetry, both naphthalene–benzene angles are 20.66 (7)°. The angle between the two naphthalene rings is 40.58 (6)°. Two hydrogen-bond rings, closed by a strong intramolecular hydrogen-bond [*d*(N···O) = 2.568 (2) Å], separate the central benzene ring from the terminal aromatic rings.

The actual nature of the tautomeric form of the title compound could not be clearly established on the bases of the C2—O1 [1.316 (2) Å] and C11—N1 [1.308 (2) Å] bond lengths. Both bond distances are intermediate between a single and a double bond, indicating the simultaneous presence of the keto-amine and the enol-imine tautomer. [The typical values of carbon-to-oxygen bond distances in quinones and phenols are 1.279 and 1.339 Å, respectively, while the corresponding carbon-to-nitrogen distances in imines and secondary amines are 1.222 and 1.362 Å, respectively (Allen *et al.*, 1987)]. In accordance with the intermediate bond lengths, the position of the H atom in the hydrogen-bonded ring could not be determined unequivocally. Its location was represented in the δF map as a large diffuse maximum of 0.29 *e*/Å^3^, positioned closer to the oxygen atom O1 than to the nitrogen atom N1. The enol to ketone tautomer ratio was determined to be 0.58 (5) : 0.42 (5) by refinement of a disordered hydrogen atom model. No significant residual electron density was detected in the area of the two disordered hydrogen atom positions in the δF map at the end of the refinement.

The peculiar bond distance scheme in naphthalene, a special arrangement of shorter and longer bond lengths is very well known (Allen *et al.*, 1987). Generally, the same naphthalene topology applies to compounds containing substituted naphthane fragments. With oxygen substitution in position 2 of naphthalene, two forms, quinoid and benzenoid (if the oxygen is protonated) can be recognised. The presence of the quionid form, *i.e.*, the quinoid effect is the cause of the quite short C3—C4 bond distance of 1.352 (3) Å in the title compound (Gavranić *et al.*,1996; Friščić *et al.*, 1998). However, there is also a slightly longer than usual C—C bond present in the naphthalene fragment. The C1—C10 bond distance is 1.452 (3) Å. The corresponding bond is 1.420 (3) Å in naphthalene. There are two possible reasons for this unusual bond distance: (i) The positions of the atoms of the enol and keto tautomer do not necessarily overlap fully, which may lead to an apparent shift of C10 closer to C9, although no anomaly in the contour δF map was observed in the region of C10 carbon atom. (ii) The possibility of electron flow to the central aromatic ring and the two highly electronegative nitrogen atoms lower the electron density in the naphthalene rings and thus weaken the aromaticity in the region of the C10 atom. Shifting of the electron density from the naphthalene core may be further assistted by the fused hydrogen-bond ring.

There are 4 molecules in the unit cell of the title compound. The molecules lie in 4(*c*) special positions of the space group with a crystallographic 2-fold axis passing through atoms C14 and C14*a*, respectively. Intermolecular interactions are dominated by van der Waals forces. There is only one very weak C—H···O contact [C13···O1^i^ 3.414 (3) Å; (i): – *x* + 3/2, *y*, *z* + 1/2]. The molecular packing framework consists of infinite layers in the (010) plane having a thickness of half of the *b*-axis (Fig. 2). Parallel layers in a stack are connected *via* extremely weak C6–H6···C6^ii^ interactions [d(C6···C6^ii^) = 3.668 (3) Å; (ii): *x* + 1/2, - *y* + 1, - *z* + 1/2]. The mutual orientation of the naphthalene rings involved in this interaction creates a herringbone motif. The herringbone motif is characteristic of planar aromatic systems (Desiraju & Gavezzotti, 1989) and is stabilised by electrostatic interactions.

### Experimental

The title compound was synthesized by applying the standard condensation procedure for the preparation of imino compounds. An ethanolic solution of 2-hydroxy-1-naphtaledehyde (10 mmol) and an ethanolic solution of *m*-phenylenediamine (5 mmol) were mixed and heated in the presence of acetic acid as catalyst. The mixture was refluxed at 331 K for 2 h. After cooling, the precipitated Schiff base was separated by vacuum filtration with a yield of 94%. A suitable single-crystal was obtained by slow recrystallization from dichloromethane solution at room temperature.

The purity of the compound was determined by NMR, FT—IR, TGA and DSC techniques. M.p. 506 K. IR / cm^-1^: 3449, 3059, 1624, 1561, 1327, 863, 825, 750. ^1^H NMR (in CDCl_3_) d/p.p.m.: 15.37 (s, 1H, OH and NH), 9.38 (s, 1H, CH=N and CH–NH), 7.11 – 8.11 (m, 9H, ar). ^13^C NMR (in CDCl_3_) d/p.p.m.: 155.26 (1 C, C=N); 146.81, 136.80, 130.67, 133.01, 129.26, 128.10, 127.30, 123,63, 121.95, 118.94, 118.11, 112,28 (12 C, ar).

### Refinement

The positional parameters and the occupancies of atoms H1 and H2, which belong to the enol and keto tautomer, respectively, were refined freely. The sum of the occupancies was constrained to 1 by useing the same *SHELXL* FVAR variable [*i.e.*, occupancies of fv(2) and 1-fv(2)]. *U*_iso_ values of both H1 and H2 were calculated as 120% of the equivalent isotropic thermal parameters of the corresponding heavy atoms. No electron density residues were observed after the refinement converged with a stable 58 (5) : 42 (5) occupancy ratio for H1 (enol tautomer) and H2 (keto tautomer), respectively.

All other hydrogen atoms were treated as riding atoms using instruction AFIX 43 (C–H 0.93 Å) with *U*_iso_(H) being 1.2 times the equivalent isotropic thermal parameter of corresponding carbon atom.

Due to now unclear reasons, at the time of data collection in 2006 the crystal-to-detector distance was set up to 70 mm. That is the likely reason that some reflections at low theta angle were not recorded.

### Crystal data

**Table:**

0.58C_28_H_20_N_2_O_2_·0.42C_28_H_20_N_2_O_2_	*F*(000) = 872
*M**_r_* = 416.46	*D*_x_ = 1.298 Mg m^−^^3^
Orthorhombic, *P**c**c**n*	Mo *K*α radiation, λ = 0.71073 Å
Hall symbol: -P 2ab 2ac	Cell parameters from 2089 reflections
*a* = 5.4292 (9) Å	θ = 4.7–26°
*b* = 26.496 (3) Å	µ = 0.08 mm^−^^1^
*c* = 14.818 (2) Å	*T* = 298 K
*V* = 2131.6 (5) Å^3^	Table, yellow
*Z* = 4	0.60 × 0.50 × 0.20 mm

### Data collection

**Table:**

Oxford Diffraction Xcalibur CCD diffractometer	1699 reflections with *I* > 2σ(*I*)
Radiation source: fine-focus sealed tube	*R*_int_ = 0.035
graphite	θ_max_ = 26.0°, θ_min_ = 4.7°
ω scans	*h* = −6→5
11523 measured reflections	*k* = −32→32
2089 independent reflections	*l* = −18→18

### Refinement

**Table:**

Refinement on *F*^2^	Primary atom site location: structure-invariant direct methods
Least-squares matrix: full	Secondary atom site location: difference Fourier map
*R*[*F*^2^ > 2σ(*F*^2^)] = 0.059	Hydrogen site location: inferred from neighbouring sites
*wR*(*F*^2^) = 0.166	H atoms treated by a mixture of independent and constrained refinement
*S* = 1.11	*w* = 1/[σ^2^(*F*_o_^2^) + (0.0822*P*)^2^ + 0.5104*P*] where *P* = (*F*_o_^2^ + 2*F*_c_^2^)/3
2089 reflections	(Δ/σ)_max_ < 0.001
153 parameters	Δρ_max_ = 0.18 e Å^−^^3^
1 restraint	Δρ_min_ = −0.15 e Å^−^^3^

### Special details

**Table:**

Geometry. All e.s.d.'s (except the e.s.d. in the dihedral angle between two l.s. planes) are estimated using the full covariance matrix. The cell e.s.d.'s are taken into account individually in the estimation of e.s.d.'s in distances, angles and torsion angles; correlations between e.s.d.'s in cell parameters are only used when they are defined by crystal symmetry. An approximate (isotropic) treatment of cell e.s.d.'s is used for estimating e.s.d.'s involving l.s. planes.
Refinement. Refinement of *F*^2^ against ALL reflections. The weighted *R*-factor *wR* and goodness of fit *S* are based on *F*^2^, conventional *R*-factors *R* are based on *F*, with *F* set to zero for negative *F*^2^. The threshold expression of *F*^2^ > σ(*F*^2^) is used only for calculating *R*-factors(gt) *etc*. and is not relevant to the choice of reflections for refinement. *R*-factors based on *F*^2^ are statistically about twice as large as those based on *F*, and *R*- factors based on ALL data will be even larger.

### Fractional atomic coordinates and isotropic or equivalent isotropic displacement parameters (Å^2^)

**Table:**

	*x*	*y*	*z*	*U*_iso_*/*U*_eq_	Occ. (<1)
O1	0.8437 (3)	0.31403 (6)	0.12718 (11)	0.0677 (5)	
H1	0.734 (12)	0.3023 (18)	0.174 (4)	0.081*	0.58 (5)
N1	0.6078 (3)	0.30360 (6)	0.27628 (11)	0.0426 (4)	
H2	0.617 (11)	0.295 (2)	0.211 (4)	0.051*	0.42 (5)
C1	0.9376 (3)	0.36355 (7)	0.25869 (12)	0.0414 (4)	
C2	0.9799 (4)	0.34832 (7)	0.16768 (13)	0.0496 (5)	
C3	1.1822 (4)	0.37011 (8)	0.11911 (14)	0.0605 (6)	
H3	1.2103	0.3604	0.0597	0.073*	
C4	1.3328 (4)	0.40449 (8)	0.15786 (15)	0.0570 (6)	
H4	1.4647	0.4172	0.1249	0.068*	
C5	1.2949 (3)	0.42177 (7)	0.24800 (14)	0.0475 (5)	
C6	1.4520 (4)	0.45830 (8)	0.28698 (16)	0.0589 (6)	
H6	1.5839	0.4707	0.2536	0.071*	
C7	1.4143 (4)	0.47577 (9)	0.37243 (16)	0.0685 (7)	
H7	1.5189	0.4999	0.3970	0.082*	
C8	1.2165 (5)	0.45689 (9)	0.42269 (16)	0.0695 (7)	
H8	1.1896	0.4689	0.4808	0.083*	
C9	1.0609 (4)	0.42084 (9)	0.38751 (14)	0.0585 (6)	
H9	0.9312	0.4089	0.4224	0.070*	
C10	1.0948 (3)	0.40169 (7)	0.29907 (13)	0.0433 (5)	
C11	0.7478 (3)	0.33924 (6)	0.30943 (12)	0.0421 (4)	
H11	0.7226	0.3492	0.3689	0.050*	
C12	0.4305 (3)	0.27711 (6)	0.32839 (12)	0.0382 (4)	
C13	0.4336 (4)	0.27599 (7)	0.42280 (13)	0.0519 (5)	
H13	0.5573	0.2925	0.4547	0.062*	
C14	0.2500	0.2500	0.46826 (19)	0.0619 (8)	
H14	0.2500	0.2500	0.5310	0.074*	
C14A	0.2500	0.2500	0.28224 (16)	0.0379 (5)	
H14A	0.2500	0.2500	0.2195	0.046*	

### Atomic displacement parameters (Å^2^)

**Table:**

	*U*^11^	*U*^22^	*U*^33^	*U*^12^	*U*^13^	*U*^23^
O1	0.0791 (11)	0.0713 (11)	0.0527 (8)	−0.0218 (9)	0.0128 (8)	−0.0176 (8)
N1	0.0444 (8)	0.0424 (8)	0.0409 (8)	−0.0046 (7)	0.0010 (7)	0.0006 (7)
C1	0.0412 (9)	0.0384 (9)	0.0444 (10)	0.0004 (7)	−0.0004 (7)	0.0018 (7)
C2	0.0543 (11)	0.0461 (10)	0.0484 (11)	−0.0017 (9)	0.0059 (9)	−0.0005 (8)
C3	0.0679 (13)	0.0622 (13)	0.0514 (12)	−0.0038 (11)	0.0165 (10)	−0.0030 (10)
C4	0.0524 (11)	0.0527 (12)	0.0659 (14)	−0.0044 (9)	0.0136 (10)	0.0083 (10)
C5	0.0412 (10)	0.0404 (10)	0.0610 (12)	0.0008 (8)	−0.0041 (8)	0.0084 (9)
C6	0.0466 (11)	0.0512 (12)	0.0789 (15)	−0.0097 (9)	−0.0091 (10)	0.0152 (11)
C7	0.0681 (14)	0.0631 (14)	0.0742 (15)	−0.0208 (11)	−0.0240 (12)	0.0047 (12)
C8	0.0804 (16)	0.0723 (15)	0.0559 (13)	−0.0186 (13)	−0.0171 (12)	−0.0048 (11)
C9	0.0616 (12)	0.0628 (13)	0.0510 (12)	−0.0142 (10)	−0.0038 (10)	−0.0007 (10)
C10	0.0405 (9)	0.0405 (9)	0.0488 (10)	0.0023 (7)	−0.0052 (8)	0.0053 (8)
C11	0.0471 (10)	0.0392 (9)	0.0400 (9)	0.0004 (8)	0.0014 (8)	−0.0022 (7)
C12	0.0400 (9)	0.0356 (9)	0.0391 (9)	0.0004 (7)	0.0019 (7)	−0.0021 (7)
C13	0.0575 (11)	0.0577 (12)	0.0404 (10)	−0.0141 (10)	−0.0053 (8)	−0.0024 (8)
C14	0.079 (2)	0.074 (2)	0.0326 (13)	−0.0218 (17)	0.000	0.000
C14A	0.0450 (12)	0.0360 (12)	0.0328 (12)	0.0018 (10)	0.000	0.000

### Geometric parameters (Å, °)

**Table:**

O1—C2	1.316 (2)	C6—H6	0.9300
O1—H1	0.96 (7)	C7—C8	1.399 (3)
N1—C11	1.308 (2)	C7—H7	0.9300
N1—C12	1.420 (2)	C8—C9	1.378 (3)
N1—H2	0.99 (6)	C8—H8	0.9300
C1—C2	1.426 (3)	C9—C10	1.417 (3)
C1—C11	1.429 (2)	C9—H9	0.9300
C1—C10	1.452 (2)	C11—H11	0.9300
C2—C3	1.434 (3)	C12—C14A	1.394 (2)
C3—C4	1.352 (3)	C12—C13	1.399 (3)
C3—H3	0.9300	C13—C14	1.386 (2)
C4—C5	1.427 (3)	C13—H13	0.9300
C4—H4	0.9300	C14—C13^i^	1.386 (2)
C5—C6	1.414 (3)	C14—H14	0.9300
C5—C10	1.427 (3)	C14A—C12^i^	1.394 (2)
C6—C7	1.364 (3)	C14A—H14A	0.9300
			
C2—O1—H1	104 (3)	C9—C8—C7	121.1 (2)
C11—N1—C12	123.14 (16)	C9—C8—H8	119.4
C11—N1—H2	120 (4)	C7—C8—H8	119.4
C12—N1—H2	117 (4)	C8—C9—C10	121.2 (2)
C2—C1—C11	119.07 (16)	C8—C9—H9	119.4
C2—C1—C10	119.47 (16)	C10—C9—H9	119.4
C11—C1—C10	121.40 (16)	C9—C10—C5	117.12 (17)
O1—C2—C1	122.40 (17)	C9—C10—C1	123.64 (17)
O1—C2—C3	118.63 (18)	C5—C10—C1	119.24 (17)
C1—C2—C3	118.94 (18)	N1—C11—C1	123.16 (17)
C4—C3—C2	121.40 (19)	N1—C11—H11	118.4
C4—C3—H3	119.3	C1—C11—H11	118.4
C2—C3—H3	119.3	C14A—C12—C13	119.18 (16)
C3—C4—C5	121.77 (18)	C14A—C12—N1	117.69 (16)
C3—C4—H4	119.1	C13—C12—N1	123.11 (16)
C5—C4—H4	119.1	C14—C13—C12	119.21 (18)
C6—C5—C10	119.87 (19)	C14—C13—H13	120.4
C6—C5—C4	121.00 (19)	C12—C13—H13	120.4
C10—C5—C4	119.12 (18)	C13—C14—C13^i^	121.8 (3)
C7—C6—C5	121.4 (2)	C13—C14—H14	119.1
C7—C6—H6	119.3	C13^i^—C14—H14	119.1
C5—C6—H6	119.3	C12—C14A—C12^i^	121.3 (2)
C6—C7—C8	119.2 (2)	C12—C14A—H14A	119.4
C6—C7—H7	120.4	C12^i^—C14A—H14A	119.4
C8—C7—H7	120.4		
			
C11—C1—C2—O1	2.9 (3)	C4—C5—C10—C9	−178.57 (18)
C10—C1—C2—O1	−179.85 (18)	C6—C5—C10—C1	−178.92 (16)
C11—C1—C2—C3	−175.43 (17)	C4—C5—C10—C1	1.1 (3)
C10—C1—C2—C3	1.8 (3)	C2—C1—C10—C9	177.18 (18)
O1—C2—C3—C4	−178.2 (2)	C11—C1—C10—C9	−5.6 (3)
C1—C2—C3—C4	0.2 (3)	C2—C1—C10—C5	−2.5 (3)
C2—C3—C4—C5	−1.6 (3)	C11—C1—C10—C5	174.74 (16)
C3—C4—C5—C6	−179.0 (2)	C12—N1—C11—C1	175.63 (15)
C3—C4—C5—C10	0.9 (3)	C2—C1—C11—N1	−0.5 (3)
C10—C5—C6—C7	−1.3 (3)	C10—C1—C11—N1	−177.74 (16)
C4—C5—C6—C7	178.7 (2)	C11—N1—C12—C14A	163.40 (14)
C5—C6—C7—C8	0.3 (3)	C11—N1—C12—C13	−18.4 (3)
C6—C7—C8—C9	0.4 (4)	C14A—C12—C13—C14	−3.2 (2)
C7—C8—C9—C10	−0.2 (4)	N1—C12—C13—C14	178.67 (14)
C8—C9—C10—C5	−0.7 (3)	C12—C13—C14—C13^i^	1.59 (12)
C8—C9—C10—C1	179.7 (2)	C13—C12—C14A—C12^i^	1.58 (12)
C6—C5—C10—C9	1.4 (3)	N1—C12—C14A—C12^i^	179.86 (16)

Symmetry codes: (i) −*x*+1/2, −*y*+1/2, *z*.

### Hydrogen-bond geometry (Å, °)

**Table:**

*D*—H···*A*	*D*—H	H···*A*	*D*···*A*	*D*—H···*A*
O1—H1···N1	0.96	1.66	2.569 (2)	154
N1—H2···O1	0.99	1.82	2.569 (2)	130
C13—H13···O1^ii^	0.93	2.67	3.413 (3)	137

Symmetry codes: (ii) −*x*+3/2, *y*, *z*+1/2.
